# Incidence of Primary Mitochondrial Disease in Children Younger Than 2 Years Presenting With Acute Liver Failure

**DOI:** 10.1097/MPG.0000000000001345

**Published:** 2016-11-23

**Authors:** Patrick McKiernan, Sarah Ball, Saikat Santra, Katherine Foster, Carl Fratter, Joanna Poulton, Kate Craig, Robert McFarland, Shamima Rahman, Iain Hargreaves, Girish Gupte, Khalid Sharif, Robert W. Taylor

**Affiliations:** ∗ Liver Unit, Birmingham Children's Hospital; † University of Birmingham; ‡ Department of Clinical IMD; § Department of Radiology, Birmingham Children's Hospital, Birmingham; || Oxford Medical Genetics Laboratories, Oxford University Hospitals NHS Foundation Trust; ¶ Nuffield Department of Obstetrics and Gynaecology, University of Oxford; # Wellcome Trust Centre for Mitochondrial Research and Highly Specialised Rare Mitochondrial Disease Service, Newcastle University; ∗∗ Genetics and Genomic Medicine, UCL Institute of Child Health, London, United Kingdom.

**Keywords:** acute liver failure, liver transplantation, mitochondrial disease, mitochondrial DNA depletion syndrome, respiratory chain deficiency

## Abstract

**Background:**

Mitochondrial liver disease (MLD), and in particular mitochondrial DNA (mtDNA) depletion syndrome (MDS) is an important cause of acute liver failure (ALF) in infancy. Early and accurate diagnosis is important because liver transplantation (LT) is often contraindicated. It is unclear which methods are the best to diagnose MLD in the setting of ALF.

**Objective:**

The aim of the study was to determine the incidence of MLD in children younger than 2 years with ALF and the utility of routine investigations to detect MLD.

**Methods:**

Thirty-nine consecutive infants with ALF were admitted to a single unit from 2009 to 2011. All were extensively investigated using an established protocol. Genes implicated in mitochondrial DNA depletion syndrome were sequenced in all cases and tissue mtDNA copy number measured where available.

**Results:**

Five infants (17%) had genetically proven MLD: *DGUOK* (n = 2), *POLG* (n = 2), and *MPV17* (1). Four of these died, whereas 1 recovered. Two had normal muscle mtDNA copy number and 3 had normal muscle respiratory chain enzymes. An additional 8 children had low hepatic mtDNA copy number but pathogenic mutations were not detected. One of these developed fatal multisystemic disease after LT, whereas 5 who survived remain well without evidence of multisystemic disease up to 6 years later. Magnetic resonance spectroscopy did not distinguish between those with and without MLD.

**Conclusions:**

Low liver mtDNA copy number may be a secondary phenomenon in ALF.

Screening for mtDNA maintenance gene mutations may be the most efficient way to confirm MLD in ALF in the first 2 years of life.

**What Is Known**

- Mitochondrial liver disease is an important cause of infantile liver failure.
- The most effective way to diagnose mitochondrial liver disease in the setting of liver failure is unclear.

**What Is New**

- Low hepatic mitochondrial DNA copy number may be a consequence of liver disease rather than a cause of liver disease.
- Screening for known mutations causing mitochondrial liver disease may be the best diagnostic strategy.

Acute liver failure (ALF) in infancy is a rare and devastating disease, which has a poor outcome without liver transplantation (LT). In approximately 20% of cases, infantile liver failure is caused by genetic mitochondrial liver disease (MLD) (1–3) with the commonest single entity being mitochondrial DNA (mtDNA) depletion syndrome (MDS).

Mitochondria contain multiple copies of mtDNA. MDS is caused by mutations in nuclear genes involved in mtDNA replication or in the maintenance of the deoxynucleotide pools required for de novo mtDNA replication, resulting in a quantitative loss of mtDNA copy number (4). Pathogenic mutations causing hepatocerebral MDS have been described in a number of genes to date with the commonest reported being *DGUOK*(5), *POLG*(6), *MPV17*(7), and *PEO1* (encoding the Twinkle helicase) (8).

Normal mitochondrial function is contingent upon the expression of many other nuclear genes, which encode constituent proteins of the respiratory chain, proteins needed for assembly of the respiratory chain, or for translation of mtDNA-encoded proteins (9). Mutations in these genes can also cause MLD and in particular mutations in *TRMU*, which encodes an enzyme essential for post-transcriptional modification of mitochondrial tRNAs, can cause infantile ALF. Such cases are particularly important to recognize because there is a significant chance of spontaneous recovery with supportive treatment (10,11).

Definitive diagnosis of most nuclear-encoded mitochondrial disorders is best established by recognizing 2 pathogenic mutations in known disease-causing genes. In the absence of an informative family history this is, however, time consuming. In the absence of a genetic diagnosis, laboratory diagnosis requires demonstrating abnormal respiratory chain function and/or loss of mtDNA copy number in clinically relevant tissue(s) (12). The most commonly sampled tissue is muscle as it is easily accessible with well-established normal ranges (12), although in multisystemic presentations of mitochondrial disease, muscle respiratory chain activities and mtDNA levels may be normal and muscle biopsy will *a priori* fail to detect isolated hepatic disease (6). Consequently liver biopsy is often necessary, if feasible. Abnormalities of respiratory chain function or of mtDNA copy number in damaged liver tissue, however, may not be due to genetic mitochondrial disease but may be a secondary change due to liver disease of other causes (13).

The aim of the present study was to determine the incidence of genetic mitochondrial disease in a group of children younger than 2 years presenting with ALF and to determine the utility of routine investigations to detect mitochondrial disease.

The present study was registered as an audit of clinical practice at Birmingham Children's Hospital NHS Trust. Children were investigated and managed according to an established in-house clinical protocol (see Supplemental Digital Content 1, Protocol).

## METHODS

Methods are available online as Supplemental Digital Content 2.

## RESULTS

A total of 39 infants (20 girls, 19 boys) presented with ALF during the study period. Ethnicity was white (30), Asian (5), and black (4). Four were born prematurely and median birth weight was 2.7 kg (range 1.8–4.1). Median age at presentation was 17 days (1–689). There had been no affected siblings in these families before the study. There were 2 sets of siblings included, 1 being twins. Three children were from consanguineous families. Overall 10 infants died without LT during the acute illness and 18 survived without LT (Table 1). Eleven underwent LT of whom 3 died in the early postoperative period. There were 2 later deaths after transplantation; 1 from progressive multisystemic disease after 3 months and 1 from vascular complications 1 year later. One child with ornithine transcarbamylase deficiency underwent elective LT 1 year after presentation because of metabolic instability and remains well 3 years later. One child (subject 37) had recurrent episodes of ALF and he was subsequently shown to have mutations in neuroblastoma amplified sequence causing recurrent ALF syndrome (14).

The results of diagnostic investigations are summarized in Table 1. The largest etiological group was infection, accounting for 12 cases, including proven HSV in 8 cases, enterovirus in 3, and adenovirus in 1. Four had inborn errors of metabolism: galactosemia (2), ornithine transcarbamylase (1), recurrent ALF syndrome (1); 4 had neonatal hemochromatosis phenotype; and 12 cases were indeterminate despite an extensive diagnostic workup.

Five children (13%) were found to have genetically confirmed MLD all of whom had MDS. All were born at full term after normal pregnancies. Three were born to consanguineous parents. Median age at presentation was 110 days (9 days to 23 months). The genetic causes were mutations in *DGUOK* (2), *POLG* (2), and *MPV17* (1).

Four of the 5 children with genetic MLD showed rapid deterioration and died within 3 weeks of presentation. One child who was homozygous for a p.(Leu304Arg) mutation in *POLG* presented at 18 months old, recovered with supportive treatment only and remains well without evidence of liver disease 6 years later.

In addition, there was 1 unexplained case (subject 24) with some features of genetic MLD. This was a female infant who became jaundiced and unwell on the first day of life. She developed progressive encephalopathy and coagulopathy with peak INR of 3.5. Muscle biopsy showed steatosis and mtDNA depletion studies were borderline in both muscle (49%) and liver (39%). Cranial magnetic resonance imaging (MRI) showed features of cerebral edema only. She underwent LT at the age of 23 days. She made an initial smooth recovery but when aged 3 months developed evidence of cardiomyopathy and died of progressive systemic disease 2 months after LT. No evidence of a genetic cause of MLD was found.

Clinical and laboratory features of the infants with genetically proven MLD compared to those with other causes of ALF are summarized in Table 2. Children with MLD tended to have lower birth weight and presented later but these differences were not significant. Similarly, there were no significant differences in the presenting laboratory values between the 2 groups. Although the median plasma lactate levels were similar between the groups, all infants with MLD had abnormal lactate values, whereas these were initially normal in 9 of 34 without MLD.

Results of tissue studies and radiology are listed in Table 1 and summarized in Table 3. Liver histology was available in 21 cases. The dominant lesion was hepatocyte necrosis in 13 cases, and this was accompanied by microvesicular steatosis in 3 cases. Including these 3 cases, significant microvesicular steatosis was present in 8 cases overall. Three who had genetically confirmed MLD had liver histology available and all showed microvesicular steatosis. The remaining 4 biopsies showed established fibrosis/cirrhosis (3) and unexplained macrophage storage material, respectively.

Liver mtDNA copy number results were available in 17 cases, 2 of whom had genetically proven MLD due to *DGUOK*. These 2 children had low (15%) and borderline (37%) liver mtDNA copy number. In 15 children without MLD, 7 had normal mtDNA copy number in liver and 8 had low levels of mtDNA: depletion (4) and borderline depletion (4). The causes of ALF in these 8 children with decreased mtDNA copy number without genetically proven MLD were indeterminate in 6 and 1 each of neonatal hemochromatosis and enterovirus infection. Two of these children died, 2 recovered without LT, and 4 underwent successful LT. One child, referred to earlier, underwent successful LT but died from apparent multisystemic disease 2 months later. None of the 5 survivors have shown evidence of multisystemic disease after up to 6 years of follow-up.

Muscle biopsies were available in 12 cases. None showed specific changes suggestive of mitochondrial involvement such as ragged-red fibers. Increased intrafiber lipid was found in 4 of 5 children with MLD who underwent muscle biopsy but was only found in 1 of 7 children without MLD. This latter child was the one who died of a multisystemic disease after LT. Muscle mtDNA copy number data were available in 11 cases, 4 of whom had MLD. Two children with MLD had low mtDNA copy number; 1 of these had complex IV deficiency in muscle tissue and 1 had normal enzyme activities. Two children with MLD had normal mtDNA copy number, and both also had normal respiratory chain activity. Six of 7 children without MLD had normal mtDNA copy number and, in the 4 cases in which these were measured, they also had normal respiratory chain enzyme activities. One had an ambiguous muscle mtDNA copy number (47%).

A total of 15 children underwent cranial MRI with diffusion-weighted imaging and 10 had magnetic resonance spectroscopy (MRS). All 5 children with MLD had MRI, which in 1 case (who had *POLG* mutation) showed symmetrical posterior midbrain changes similar to those reported in mitochondrial disease (4). Three showed cerebral edema which had a cytotoxic or demyelination pattern in 2 cases and a vasogenic pattern in 1. Two children had an initial normal MRI, but in 1 case repeat MRI showed progression to vasogenic cerebral edema. Ten children without MLD had an MRI, which was normal in 3 and showed cerebral edema in 7, appearing cytotoxic in 2 and vasogenic in 5. Five children with MLD had MRS, which showed a lactate peak in 3. Five children without MDS had MRS, which showed a lactate peak in 2.

## DISCUSSION

Infantile ALF is a serious disorder with a variety of potential causes. A structured, rapid approach to diagnostic investigations in tandem with identifying and treating correctable disorders is necessary. We have confirmed that MLD is an important cause of infantile ALF and that genetically confirmed MDS is the commonest entity in this group. The outlook for affected infants is poor and early recognition is important to minimize unnecessary invasive investigations, to prevent inappropriate LT, and to facilitate family counseling. Ideally, diagnostic investigations should be available within days of presentation. The definitive method to diagnose MLD is by detection of 2 pathogenic mutations in recognized genes; hence, some attempt at targeted mutation detection should be initiated at the time of initial presentation. This could later be reassessed if other diagnostic information becomes available.

In the absence of pathogenic disease-causing mutations, the diagnosis of MLD depends on tissue studies. It has been a *sine qua non* in the investigation of suspected mitochondrial disease that an affected tissue should be studied. Our findings cast doubt on this approach in the setting of ALF. We have found that reduced mtDNA copy number in affected liver tissue is not synonymous with genetically proven MDS. In 3 of the 8 cases reported here plausible alternative causes of ALF were found. In the 5 unexplained cases we cannot definitely exclude mitochondrial disease because undetected genetic disorders may yet be present. For at least 4 of these cases, primary mitochondrial disease, however, seems unlikely; no pathogenic mutations have been detected and no other evidence to support progressive mitochondrial disease has appeared even after prolonged follow-up. One of these children, who developed a multisystemic disease after LT, did have some features of systemic mitochondrial disease but no genetic cause was detected.

There have been few studies examining the accuracy of low hepatic mtDNA copy number to diagnose MLD in which the primary presentation is with clinical liver disease. In end-stage liver disease some studies have shown that low mtDNA copy number appeared to be specific for MDS (15), but in another study 10 of 45 unselected cases undergoing LT had low copy number (16). Low mtDNA copy number has also been reported in Mauriac syndrome in which the clinical findings are often reversible (17). In ALF low copy number appears to be common irrespective of the etiology. Helbling et al (15) found low mtDNA copy number in 29 of 44 patients with ALF and all 3 cases reported by Lane et al (16) had decreased number. Decreased copy numbers were found even where a plausible nonmitochondrial cause of ALF existed. In contrast, Al-Hussaini and colleagues (1) found hepatic mtDNA copy number to be specific for MDS, but only 4 children in whom liver disease did not have a mitochondrial cause were studied.

Overall these reports are consistent with our findings and suggest that liver disease, and especially ALF, may cause a secondary lowering of mtDNA copy number as a consequence of the primary disease. We cannot exclude that as yet undetected mutations in other genes underlie these examples of mtDNA depletions. An important part of the present study is, however, the length of subsequent follow-up, which makes late sequela of unrecognized disease less likely. We also cannot comment as to whether the low mtDNA copy number contributes to the pathogenesis of ALF in these cases. What we can say is that clinical management decisions, including whether to proceed with transplantation, should not be influenced by hepatic mtDNA copy number in the absence of proven mutations.

Rapid detection of pathogenic mutations in candidate genes remains the ideal method for diagnosis of MLD. The commonest causes of MLD are recessively-inherited mutations in *DGUOK*, *POLG*, *MPV17*, *PEO1*, and *TRMU*(1,7,18). Certainly, screening for mutations in these genes should be initiated at presentation with infantile ALF. The prioritization of genes to screen will depend on local experience and available facilities, while recognizing that this approach will only recognize a proportion of defects.

Up to 1300 nuclear genes encode mitochondrial-related proteins and the basis of many defects remain unknown (18). It is to be hoped that next-generation screening techniques, including custom captures of specific nuclear-mitochondrial genes or whole exome or whole genome sequencing, will transform this situation. For example, it is now possible to sequence the entire mitochondrial genome and all coding exons of the nuclear genes encoding mitochondrial proteins. Initial experience using this approach for children with suspected mitochondrial disease achieved a firm diagnosis in 24% of cases and a probable cause in a further 30% (19). The major future challenge will be to ensure next-generation screening results can be made available in a clinically relevant timescale, that is, within days if possible, and certainly within a fortnight, although this will vary according to local practice and laboratory diagnostic algorithms.

Even establishing a molecular diagnosis does not absolutely establish prognosis. Although 4 of the 5 cases showed rapid progression and death from systemic disease, 1 child with recessive *POLG* mutations recovered spontaneously; interestingly, she was homozygous for the p.(Leu304Arg) mutation that is usually associated with a late-onset *POLG* phenotype of sensory ataxic neuropathy with dysarthria and ophthalmoplegia rather than liver disease (20). This mutation has been reported to cause ALF in compound with a second (p.[Ala467Thr]) heterozygous *POLG* mutation (21), which supports the observation of Tzoulis et al (22) that compound heterozygosity often has a worse prognosis than homozygous *POLG* mutations. Recent work has suggested that the pattern of mtDNA when visualized by fluorescence microscopy in cultured fibroblasts may also provide further prognostic information (12). Spontaneous recovery from ALF has been previously recognized in at least 1 other child with *POLG* mutations (23) and emphasizes that, although LT is inappropriate in this group, these patients should not be denied appropriate supportive treatment.

Recognizing and defining central nervous system involvement in MLD is crucial to guide prognosis and management. In ALF from other causes central nervous system involvement with encephalopathy is common and is generally reversible after successful LT. In MLD such involvement, however, may be a contraindication to LT. MRI abnormalities are common, but not invariable, in MLD and range from widespread generalized white matter changes to cortical atrophy to specific involvement of deeper brain structures (1,4,24). These latter appear to be more specific for MLD but were found in only 1 of our cases. We found that generalized abnormalities were common in ALF irrespective of cause and that there was a similar distribution between cytotoxic and vasogenic cerebral edema whether or not liver failure was due to MLD. Similarly, MRS detection of a lactate peak did not provide useful discrimination between mitochondrial and nonmitochondrial causes. We did confirm that MRI changes may develop and evolve quickly and that serial evaluation may be necessary. In this group of ill infants MRI, however, only helped the decision on appropriateness of LT in a small proportion of cases.

In conclusion, we have shown that MLD is an important cause of infantile ALF and that mutation detection is the most robust diagnostic method.

## Supplementary Material

Supplemental Digital Content

## Supplementary Material

Supplemental Digital Content

## Figures and Tables

**TABLE 1:** Investigations and clinical outcome of 39 infants with acute liver failure admitted to Birmingham Children's Hospital from 2009 to 2011

Subject no.	Diagnosis	Age at presentation, days	Initial serum lactate, mmol/L	Muscle histology	Muscle respiratory chain activity	Muscle mtDNA copy number, %	Liver histology	Liver mtDNA copy number, %	Cranial MRI	MR spectroscopy	Mutations detected	Outcome
1	MDS (*DGUOK*)	9	7.2	Increased lipid	Decrease in complex IV	45.0	Micro- and macrovesicular steatosis	37.0	Vasogenic pattern cerebral edema	Lactate peak	p.[Glu44Lys]; [Glu44Lys]	Died
2	MDS *(DGUOK)*	16	25.0	Increased lipid	Normal	50.0	Hepatocellular necrosis and microvesicular steatosis	15.0	Vasogenic pattern cerebral edema	Lactate peak	p.[Arg39X]; [Arg39X]	Died
3	MDS (*MPV17*)	110	9.9	Increased lipid	Decrease in complexes I–IV	ND	ND	ND	Normal	Normal	p.[Gln44X]; [Gln44X]	Died
4	MDS (*POLG*)	477	6.0	Normal	Normal	55.0	ND	ND	Cytotoxic pattern cerebral edema	Lactate peak	p.[Thr914Pro]; [Ala467Thr]	Died
5	MDS (*POLG*)	503	3.7	Increased lipid	Normal	107.0	Micro- and macrovesicular steatosis	ND	Post-midbrain lesion in keeping with mitochondrial disease	Normal	p.[Leu304Arg]; [Leu304Arg]	Recovered. Remains well 6 years later.
6	HSV	8	8.5	ND	ND	ND	Hemorrhagic necrosis	83	ND	ND	None	Died following LT
7	HSV	11	16.0	ND	ND	ND	Hemorrhagic necrosis	ND	ND	ND	None	Died
8	HSV	8		ND	ND	ND	ND	ND	ND	ND	None	Recovered
9	HSV	8		ND	ND	ND	ND	ND	ND	ND	None	Recovered
10	HSV	11		ND	ND	ND	ND	ND	ND	ND	None	Recovered
11	HSV	11		ND	ND	ND	ND	ND	ND	ND	None	Died following LT
12	HSV	21	6.4	ND	ND	ND	ND	ND	ND	ND	None	Died
13	HSV	17	4.7	ND	ND	ND	ND	ND	Vasogenic pattern cerebral edema	ND	None	Recovered
14	Enterovirus	6	22.4	ND	ND	ND	ND	ND	ND	ND	None	Died
15	Enterovirus	12		Normal	Normal	54.0	Hemorrhagic necrosis	34.0	ND	ND	None	Died following LT
16	Enterovirus	9	9.6	ND	ND	ND	ND	ND	Vasogenic pattern cerebral edema	Lactate peak	None	Died
17	Adenovirus	112		ND	Normal	ND	ND	ND	Cytotoxic pattern cerebral edema	ND	None	Recovered
18	Indeterminate	498	5.6	ND	ND	ND	Panacinar necrosis	29.0	ND	ND	None	Successful LT
19	Indeterminate	228	3.3	ND	ND	ND	Microvesicular steatosis	25.0	ND	ND	None	Recovered
20	Indeterminate	52	3.8	ND	ND	ND	Severe fibrosis and microvesicular steatosis	40.0	ND	ND	None	Recovered
21	Indeterminate	349	4.7	ND	ND	ND	Panacinar necrosis and macrovesicular steatosis	42.0	ND	ND	None	Successful LT
22	Indeterminate	260	1.2	ND	ND	ND	ND	ND	ND	ND	None	Died
23	Indeterminate	15	4.3	Normal	Normal	127.0	Hepatocyte necrosis and macrophage storage material	91.0	Normal	ND	None	Died
24	Indeterminate	1	3.0	Increased lipid	ND	47.0	Panacinar necrosis	39.0	Vasogenic pattern cerebral edema	ND	None	Underwent LT but died of progressive multisystemic illness 2 months later
25	Indeterminate	689	3.8	ND	ND	ND	Hepatocellular necrosis and microvesicular steatosis	60	ND	ND	None	Recovered
26	Indeterminate	644	2.7	ND	ND	ND	Panacinar necrosis	92	ND	ND	None	Recovered
27	Indeterminate	1	2.1	Normal	Normal	83.0	ND	ND	ND	ND	None	Recovered
28	Indeterminate	37		Normal	Normal	81.0	Moderate fibrosis	ND	Normal	ND	None	Recovered
29	Indeterminate	216	4.4	ND	ND	ND	Panacinar necrosis	25	Cytotoxic pattern cerebral edema	Normal	None	Successful LT but died 1 year later from vascular complications
30	NH	8	4.7	ND	ND	ND	ND	ND	ND	ND	None	Recovered
31	NH	24	3.2	Normal	ND	ND	Panacinar necrosis	65.0	ND	ND	None	Successful LT
32	NH	21	0.7	ND	ND	ND	Established cirrhosis	54.0	ND	ND	None	Successful LT
33	NH	13	1.0	Normal	Normal	88.0	Panacinar necrosis	26.0	Cytotoxic pattern cerebral edema	Normal	None	Successful LT
34	OTC deficiency	599	2.5	ND	ND	ND	ND	ND	ND	ND	None	Recovered. Underwent elective LT age 3.
35	Galactosemia	7		ND	ND	ND	ND	ND	ND	ND	None	Recovered
36	Galactosemia	9		ND	ND	ND	ND	ND	ND	ND	None	Recovered
37	RALF	539	5.8	ND	Normal	80	Microvesicular steatosis	127	Normal	Normal	None	Recovered and had subsequent recurrent bouts of liver failure
38	HLH	6	4.3	ND	ND	ND	ND	ND	Vasogenic pattern cerebral edema	Lactate peak	None	Died
39	Chemotherapy	121	6.6	ND	ND	ND	Macro- and microvesicular steatosis	ND	ND	ND	None	Recovered

HLH = hemophagocytic lymphohistiocytosis; HSV = herpes simplex virus infection; LT = liver transplantation; MDS = mitochondrial depletion syndrome; MRI = magnetic resonance imaging; MRS = magnetic resonance spectroscopy; mtDNA = mitochondrial DNA; ND = not done; NH = neonatal hemochromatosis phenotype; OTC = ornithine transcarbamylase deficiency; RALF = recurrent acute liver failure.

**TABLE 2:** Clinical and laboratory features in infant with and without genetically proven mitochondrial liver disease as a cause of liver failure

	Genetically proven MLD (n = 5)	Other causes of ALF (n = 34)	*P*
Birth weight, kg	2.6 (2.3–2.9)	2.8 (1.8–4.1)	0.76
Age at presentation, days	110 (9–503)	16 (1–689)	0.33
Prothrombin time, s	26 (23–41)	34 (18–120)	0.5
Serum bilirubin, μmol/L	113 (34–335)	146 (5–492)	0.79
ALT, IU/L	113 (42–1875)	940 (19–6000)	0.11
Lactate, mmol/L	7.2 (3.7–25)	4.3 (0.7–22.4)	0.11

ALF = acute liver failure; ALT = alanine aminotransferase; MLD = mitochondrial liver disease.

**TABLE 3:** Results of tissue studies and radiology undertaken in infants with and without genetically proven mitochondrial liver disease

	Genetically proven MLD (n = 5)	Other causes of ALF (n = 34)
Liver mtDNA depletion	2/2	8/15
Muscle mtDNA depletion	2/4	1/7
Abnormal muscle respiratory chain enzymes	2/5	0/4
Muscle steatosis	4/5	1/7
Hepatic steatosis	3/3	5/18
MRS lactate peak	3/5	2/5

ALF = acute liver failure; MLD = mitochondrial liver disease; MRS = magnetic resonance spectroscopy; mtDNA = mitochondrial DNA.
