# Supplementary material for: Incidence of Primary Mitochondrial Disease in Children Younger Than 2 Years Presenting With Acute Liver Failure
Source: J Pediatr Gastroenterol Nutr. 2016 Nov 23;63(6):592–7. doi: 10.1097/MPG.0000000000001345 (PMC5113754; doi:10.1097/MPG.0000000000001345)
Supplement: Supplemental Digital Content [file jpga-63-0592-s001.docx]

ACUTE LIVER FAILURE

MANAGEMENT PROTOCOL

BACKGROUND

Acute liver failure is a rare life threatening event. In children it is defined as a multisystemic disorder in which severe impairment of liver function, with or without encephalopathy, occurs in association with hepatocellular necrosis, reflected as liver synthetic failure in a child with no previously recognized liver disease.

Early recognition and prompt referral to a specialist centre with a multidisciplinary team including hepatologists, intensivists, specialist nurses and surgeons with access to liver transplant facilities is mandatory.

Causes of acute liver failure vary with age and in ~ 45%iof children remain unknown despite thorough investigation. Outcome is unpredictable in individual cases, but prognosis and need for liver transplantation may be assessed by the following parameters.

Poor prognostic factors:

1. Age <10 years
2. Infants < 1yr with severe coagulopathy secondary to metabolic liver disease or familial erythrophagocytosis
3. Seronegative hepatitis
4. severe coagulopathy (PT>55secs)
5. prolonged duration of illness before onset of hepatic encephalopathy
6. Degree of encephalopathy:
   - Grade I-II : 44% mortality
   - Grade III-IV : 78% mortality
7. Shrinking liver size
8. Associated renal failure

##### Other facts:

- Overall mortality: without liver transplant : >70%

With liver transplant : 25 - 30%

- Children with ALF secondary to Hepatitis A infection, autoimmune hepatitis or paracetamol overdose are more likely to recover spontaneously if appropriately treated.
- Spontaneous recovery from acute liver failure is associated with histological and biochemical recovery, even when extensive necrosis is present.

TELEPHONE REFERRAL

Non paracetamol acute liver failure

- Vitamin K
- 6 hourly clotting, pH, creatinine, glucose and assessment for encephalopathy

Paracetamol induced Acute Liver Failure

- Parvolex
- Vitamin K
- 6 hourly clotting, ph, creatinine, glucose and assessment for encephalopathy

INR <2

Manage locally unless acidosis, renal dysfunction or encephalopathy

INR >2

Admit

Inform ITU consultant

Admit if: INR > 4 and/or renal dysfunction and/or pH<7.3 and/or co-ingestion

Inform ITU consultant

INR <2

Consider admission

EVALUATION ON ADMISSION

Full history including information on:

- IV injections, needles and needlestick injury
- ? blood products
- Foreign travel
- Contact with jaundice/liver conditions
- Family history of liver disease
- Consanguinity (?metabolic disorder)
- Full pregnancy history (jaundice/pruritus 🡪?PFIC; FLP/HELLP 🡪? FAO defect)
- Previous sibling death (?tyrosinaemia, ?NNH)
- Sexual contacts
- Parents drug habits and medications, lifestyle drugs incl. alcohol
- Patients medications or other suspect poisons (e.g. mushrooms, herbal remedies)
- Contact with fresh water and/or animals

### Full Clinical Examination:

- Pay particular attention to and record degree of encephalopathy (see page 10 )
- State of hydration
- Evidence of spontaneous bleeding
- Evidence of chronic liver disease
- Mark upper and lower margins of liver onto abdomen with waterproof marker
- Spleen size
- Ascites

**INVESTIGATIONS**

# BLOODS

# Clinical Chemistry

**a) random**

Acid/Base + gases

Ammonia (inform lab by ‘phone)

Bilirubin (total and unconjugated), AST, ALT, GGT, ALP, Total protein, albumin, urea, Creatinine, sodium, phosphate, potassium, calcium, magnesium,

plasma osmolality,

Follow ICE guidelines for blood amounts

Fe/TIBC

Galactosaemia/Tyrosinaemia screen

Alpha 1 antitrypsin level + phenotype

Cu/Caeruplasmin + penicillamine challenge if indicated

Paracetamol + salicylates (if suspected)

AFP

amylase

Acyl carnitines profile – (can be sent as plasma)

LDH

CK – if indicated

Plasma for quantitative amino acids

# b) fasting

Cortisol

Glucose

Lactate

3-hydroxy-butyrate

Free fatty acids

# Immunology

Autoantibodies (LKM, SMA, ANA, GPC, AMA)

Complements (C3, C4)

Immunoglobin (IgG, IgM, IgA)

# Haematology

PT

APPT

Fibrinogen

Full blood count & differential

Reticulocytes

Group + Save

Coombs test

Ferritin

# Microbiology

# Serology

# Hepatitis A IgM Hepatitis B surface Ag

# Hepatitis B anti-core IgM Hepatitis C-Anti HBC

Hepatitis E + PCR
CMV - IgG/M
EBV - IgG + PCR

+ save serum

Consider : *Leptospira* - if fresh water contact
*Parvovirus PCR* – if features of infection - erythema infectiosum, arthritis or aplastic anaemia
*Bartonella henselae, Coxiella burnetii* serology –if animal contact
**Neonates** - consider *syphilis* serology, *coxsackie* serology and *enteroviruses* (ECHO) stool sample + viral culture

**Every patient with acute liver failure should be discussed with Dr Jim Gray on the day of admission (if possible)**

**6 Molecular Genetics**

Collect DNA from infants

# URINE

## Clinical Chemistry:

Succinylacetone

3 universal containers

10-20mls

Aminoacids

Organic acids

Save urine for further tests (e.g. bile salts, orotic acid, oligosaccharides)

**Toxicology screen – important to catch first urine**

Reducing substances if indicated

TRP/calcium/sodium/osmolality, pH if indicated

Protein/Creatinine

Ward tests with dipstick pH, glucose, ketones etc + reducing substance

24 hour urine collection with Penicillamine challenge at 24 hours, 36 hours if indicated. (acid wash bottle for clinical chemistry)

# Microbiology

M+C/S

CSF (only if indicated and after d/w consultant):

Glucose

Lactate

Total protein (if indicated)

# STOOLS:

Stools for virology (e.g. Echovirus, Adenovirus) (see under serology)

# RADIOLOGY:

- **Abdominal ultrasound** for liver, spleen size, vessel size, direction of flow etc.
- **MRI abdomen** if neonatal haemochromatosis is suspected (may require general anaesthesia)

  On T2-weighted (T2-W) sequences tissues with increased iron content have low signal intensity. In NH the reticuloendothelial system is spared so that the spleen retains a normal, higher signal intensity compared with affected tissues. Skeletal muscle is used by some authors as a ‘reference’ for tissue signal intensity, tissues with increased iron content having a lower signal intensity compared with skeletal muscle. Furthermore the signal intensity of the pancreas in these case reports varies from slightly lower than that of the spleen, to low signal intensity comparable with the liver, to very low signal (black). It is also important to be aware that siderosis or increased iron content of the liver is physiological in the third trimester of pregnancy and in the neonatal period so that the liver will have a lower signal intensity than the spleen or skeletal muscle on T2-W sequences even in normal neonates.
- **Chest x-ray** if indicated
- **Cranial CT** – only indicated if suspected intracranial bleed – discuss with consultant.

# NEUROPHYSIOLOGY

EEG for baseline

# HISTOPATHOLOGY

Lip biopsy if indicated (? NNH)

Muscle biopsy if indicated (? Mitochondrial) – surgical SpR to liaise with Pathology lab in advance

Bome marrow trephine/aspirate – if indicated (? HLH, ? thesaurismoses)

Consider liver biopsy, ? transjugular liver biopsy if persistent coagulopathy (histoology + E.M + frozen sample)

# ASSESSMENT OF ADRENAL FUNCTION

Adrenal dysfunction is common in adult acute liver failure and may contribute to metabolic and haemodynamic instability. It is more frequent in patients with severe liver dysfunction.

(Harry et al., HEPATOLOGY; 2002; 36: 395-402).

Low-dose Synachten Test - Indication to be discussed with consultant-see Protocols folder on M:\ drive for test procedure

# OTHER

- **ECG + Echo** if indicated to assess for systemic disease or as part of transplant assessment – discuss with consultant.
- **Skin biopsy** (in culture medium) for freezing - after discussion with consultant.

INPATIENT MANAGEMENT PROTOCOL

Medical management

Successful management requires a multidisciplinary approach with close liaison between paediatricians, surgeons, intensivists, anaesthesists, dietitians and nurses. A daily decision should be made as to whether intensive care monitoring is required.

**General Management:**

Management is directed towards:

1. Hepatic support ) whilst awaiting
2. Prevention and treatment of complications ) recovery or
3. Early consideration for liver transplantation ) suitable donor.

**Referral to PICU**:

- - Every child with ALF should be discussed between consultant hepatologist and consultant intensivist on admission.
  - A child with grade 2 encephalopathy should be reviewed by consultant intensivist and a joint decision with consultant hepatologist should be made re PICU transfer.
  - A child with grade 2 encephalopathy requiring additional procedures (line insertion, endoscopy, muscle biopsy etc.) may need earlier PICU admission, this should be discussed between the consultant hepatologist and consultant intensivist*.
    NOTE: Children should initially be managed without CVL for as long as possible*
  - Grade 3 encephalopathy is an indication for transfer to PICU

| 1 | **No sedation** (except for procedures!!) | Nb. Sedation masks encephalopathy! |
| --- | --- | --- |
| 2 | Minimal handling |  |
| 3 | Consider Central Venous access | D/W Liver Unit Anaesthetists |
| 4 | **Regular PICU Monitoring**:   - Heart and respiratory rate, ECG monitor - Cutaneous oximetry - Arterial BP (on ITU only) - CVP (4-8 mmHG) - Core/toe temperature - Neurological observations, baseline EEG - Gastric pH (>5.0) - Urine output (by catheter preferred) - Blood glucose/BM monitoring (> 4 mmol/L) - Acid-base balance, lactate - Electrolytes (incl Mg, Ca and Phosphate), ammonia - ALP, AST, ALT, GGT, SBR, Alb - Coagulation screen - Plasma and urine osmolality - If possible a retrograde jugular venous catheter should be inserted for assessment of cerebral oxygen extraction | )  ) at least 4-hourly, or as  ) indicated  )  )  )  6-hourly  Aim: 0.5-2 mls/kg/hour  8-hourly  8-hourly  Twice daily  Daily  8-hourly  Daily (more frequently if indicated) |
| 5 | **Fluid management:**   - Fluid balance 50-75% maintenance - Dextrose ivi (10-50%) - Maintain circulating volume with colloid (4.5% or 20% Human albumin solution) - Sodium (0.5-1 mmol/kg/day) - Potassium (2-4mmol/kg/day) - Consider mannitol (see encephalopathy) - Consider early Haemofiltration or MARS | Twice daily  Depending on CVP and BP  Aim for Blood glucose level: 4 - 8  Correct any electrolyte imbalance |
| 6 | Drugs | See ‘baseline drugs’ |
| 7 | **Nutrition :**   - NBM until galactosaemia, tyrosinaemia, urea cycle disorder ruled out - Appropriate enteral feed – discuss with dietitian/metabolic dietitian - Consider PN | Protein: 1-2 g/kg/day |
| 8 | Coagulation support | Daily Vitamin K (i/v)  See coagulopathy section |

# BASELINE DRUGS

| 1 | Vitamin K | < 1 year 2.5 mg/dose OD i/v  > 1 year 5 mg/dose OD i/v  >10 year 10 mg/dose OD i/v |
| --- | --- | --- |
| 2 | Antacids | Ranitidine 1-3 mg/kg/dose TDS i/v  Or  Omeprazole 0.5 mg/kg/dose BD i/v or orally  Sucralfate 250-500 mg/dose QDS (if PH <5 after H_2_-antagonist or PPI) |
| 3 | Lactulose | 2-4 mls/kg/dose TDS |
| 4 | *N*-acetylcysteine | 150 mg/kg/day continuous infusion (only if paracetamol OD or NAC study) |
| 5 | **Broad-spectrum antibiotics:**   - Tazocin - Metronidazole | 90mg/kg/dose tds  Double Click on word icon to view  8 mg/kg/dose TDS i/v (bd for neonates upto 1/12) |
| 6 | **Antifungals:**   - Fluconazole     or   - L- Amphotericin (Ambisome) | 3-6 mg/kg/day i/v  **Neonate under 2 weeks**  3-6 mg/kg on first day then 3mg/kg every 72 hours  **Neonate 2-4 weeks**  3-6 mg/kg on first day then 3mg/kg every 48 hours  3 mg/kg/day i/v |
| 7 | **Antiviral treatment:**   - Aciclovir - Must be started in all infants | < 3 mths : 10 mg/kg TDS i/v  3 mths-12 years : 250 mg/m^2^ TDS i/v  >12 years : 5 mg/kg TDS i/v  nb. Double the dose in immunocompromised or severe illness |

**MANAGEMENT OF MEDICAL PROBLEMS/COMPLICATIONS:**

1. Hypoglycaemia
2. Coagulopathy and Haemorrhage
3. Encephalopathy/Raised Intracranial Pressure
4. Convulsions
5. Renal dysfunction
6. Cardiovascular problems
7. Metabolic Acidosis

Where multiple complications occur the need for intensive care monitoring should be reviewed even where each individual complication might not warrant this in isolation.

1. ***HYPOGLYCAEMIA:***

General: - severe hypoglycaemia (blood glucose < 3.5 mmol/L) is common

- may contribute to CNS impairment and other organ dysfunction

- refractory hypoglycaemia carries a poor prognostic implication

Management:

- minimal 4-hourly BM monitoring
- intravenous glucose administration (10-50% dextrose)
- avoid hyperglycaemia

1. ***Coagulopathy and haemorrhage:***

General:

- Profound coagulopathy can develop secondary to:
  - - failure of hepatic synthesis of clotting and fibrinolytic factors
    - reduction in platelet numbers (depending on diagnosis)
    - intravascular coagulation (if sepsis present)
- Prothrombin time (PT) is the most sensitive measure of hepatic synthesis of clotting factors.

Management:

- Daily dose of intravenous Vitamin K
- Do not routinely correct coagulopathy with blood products (eg FFP or cryoprecipitate) as PT is a sensitive guide to prognosis and need for liver transplantation.
- Once decision to list for transplant has been made start correcting PT > 40secs (increased risk of bleeding).
- Use FFP (10-15 ml/kg every 6 hours), cryoprecipitate and platelets (if indicated)
- Consider use of Recombinant factor VIIa (rFVIIa) – consult Haematologist
- Haemofiltration may be required to control fluid balance (d/w ITU team)

1. ***eNCEPHALOPATHY/: raised intracranial pressure***

General:

- Deranged cerebral function associated with hepatic failure
- Possible causes include:

1. accumulation of toxic substances and toxic damage of the brain
2. rising intracranial pressure (cerebral oedema) secondary to

- Fluid overload from therapeutic efforts to correct coagulopathy and hypotension
- Failure to maintain blood glucose concentrations
- Failure to maintain systemic blood pressure (cerebral ischaemia)
- Brain death associated with cerebral oedema is the commonest cause of death in fulminant liver failure
- Prognosis is poor once it is evident
- May be exacerbated by sepsis, GI bleeding, electrolyte disturbances, etc
- Children may fluctuate rapidly from one stage to the other
- Suspected raised intracranial pressure alone is ***not*** an indication for a cranial imaging (CT)
- ICP monitoring (“bolts”) may be used on occasion and the decision will need to be made on a case by case basis.

Clinical stages of hepatic encephalopathy:

| ***Stage*** | ***Asterixis*** | ***EEG changes*** | ***Clinical manifestations*** |
| --- | --- | --- | --- |
| **I**  **(prodrome)** | Slight | Minimal | Mild intellectual impairment, irritable, lethargy/mildly obtunded, disturbed sleep-awake cycle |
| **II**  **(impending coma)** | Easily elicited | Generalized slowing of rhythm | Drowsiness, confusion, inappropriate/odd behaviour, disorientation/not recognizing parents, mood swings, photophobia |
| **III**  **(stupor)** | Present if patient co-operative | Grossly abnormal slowing | Unresponsive to verbal commands, markedly confused, aggressive, delirious, hyperreflexia, positive Babinski sign |
| **IV**  **(coma)** | Usually absent | Delta waves, decreased amplitudes | Unconscious, initial response to pain present, later decerebrate or decorticate response to pain present or absent, areflexia |

Management stage I and II:

- Every effort should be made to prevent cerebral oedema!!
- Nurse child with head elevated at 20^0^ and no neck flexion (to decrease ICP and minimize cerebral irritability)
- Maintain oxygenation
- Restriction of dietary protein
- Carefully review fluid balance:
  - fluid restrict to 50-75% maintenance (depending on CVP)
  - maintain urine output ~ 0.5-2 mls/kg/hour
- Minimize formation of nitrogenous substances by the intestine:
  - - Lactulose (2-4 mls/kg/dose TDS)
- Avoid sedation (this may mask encephalopathy):
  - - Sedation should only be given in ITU
    - Use short-acting barbiturates or opiates
    - Avoid benzodiazepines

All children who develop grade II encephalopathy should be discussed with ITU consultant and hepatology consultant. Indications for transfer to ITU should be agreed.

Management stage III and IV:

- All children with Grade III encephalopathy should be admitted to ITU for elective ventilation.
- As management of stage I and II
- CT-scan: Consider if focal cerebral lesion (i.e. bleeding) suspected
- Give Mannitol : 0.5-2 gram/kg over 1 hour (7mls/kg of 20% mannitol)

Repeat every 6-8 hours for a maximum of 48 hours

Measure osmolarity every 12 hours (max 310 mosmol/kg)

- Elective ventilation
- Reassess need for ventilation after 72 hours
- Consider insertion of retrograde jugular venous catether
- Measure jugular venous saturation 4 hourly
- Intracranial pressure monitoring:
  - Indications for this should be reviewed on a case by case basis
- May need to consider Thiopentone : 4-8 mg/kg stat i/v

0.5-3 mg/kg/hour infusion

(CFAM monitoring is desired but not essential during Thiopentone infusion to maintain burst suppression)

1. ***Convulsions:***

General:

- Clinical presentation may be atypical or occult in children
- May be caused by: underlying cause of ALF (toxic injury, viral, metabolic, etc)

Electrolyte imbalance

Cerebral oedema

Management:

- Correct electrolyte imbalance (if present)
- Carefully review fluid balance
- Consider Mannitol infusion if caused by possible cerebral oedema and plasma sodium less than 135 mmol/L:

0.5-2 gram/kg over 1 hour (2.5 - 10mls/kg of 20% mannitol)

Repeat every 6-8 hours for a maximum of 48 hours

Measure osmolarity every 12 hours (max 310 mosmol/kg)

- See Appendix A for management of status epilepticus

1. ***RENAL DYSFUNCTION:***

General:

- Defined as: urine output < 0.5 ml/kg/hour in 2 consecutive hours
- Possible causes: Hepato-renal syndrome

Dehydration

Low CVP/Low cardiac output

Management:

- Colloid challenge : 10-20 mls/kg over 30-60 mins; repeat if no response
- If CVP is high (> 8 mmHG) : start renal dose of dopamine 2-5 μg/kg/min
- If no response : start Frusemide : 1-2 mg/kg stat i/v
- Consider Terlipressin (=Glypressin):

Children *12-18 years*: 2mg stat, then 6-8 mg/24h

- If established renal failure:
  1. Frusemide infusion : 0.25-1 mg/kg/hour
  2. consider Haemofiltration early (liaise with ITU/renal unit)
  3. Consider MARS (see Appendix B)

1. ***CARDIOVASCULAR PROBLEMS/low cardiac output:***

General:

Consider following causes: hypovolaemia

Hypoxia

Hypoglycaemia

Sepsis

Management:

- Colloid challenge : 10-20 mls/kg over 30-60 mins
- Inotrope support should be discussed with PICU staff

1. ***Metabolic acidosis***

General:

Consider following causes: Hypovolaemia

Hypoxia

Sepsis

Renal failure

Management:

- Generally TREAT if base excess (BE) >10 and pH <7.25
- Give: 8.4% Sodium Bicarbonate intravenously as follows:

mls bicarbonate = weight (kg) x base deficit (i.e. half correct)

6

1. ***SEPSIS***

General:

Signs of sepsis may be subtle, e.g. rise in heart rate or core-toe temperature

gradient, fall in blood pressure or urine output, hypo- or hyperglycaemia,

hypothermia, deterioration in mental state, fits, increasing acidosis.

Management:

- Do full septic screen, omitting LP and supra-pubic puncture.
- Start broad spectrum antibiotics and antifungals ‘’blind’’ (Cefuroxime, Amoxicillin and Metronidazole), see dose under “baseline drugs”
- escalate after discussion with microbiologist

1. **Other therapies:**
2. MARS therapy (see Appendix B)
3. Liver Transplantation

**APPENDIX A:**

### ALGORITHM FOR MANAGEMENT OF STATUS EPILEPTICUS

AIRWAY

High flow oxygen

Don’t ever forget glucose

VASCULAR ACCESS?

Yes No

LORAZEPAM DIAZEPAM

0.1 mg/kg IV/IO 0.5 mg/kg PR

10 Minutes 10 Minutes

Yes

LORAZEPAM VASCULAR ACCESS?

0.1 mg/kg IV/IO

10 Minutes No

PARALDEHYDE

0.4 ml/kg PR

i.e. 0.8 ml/kg of prepared solution

PHENYTOIN

18 mg/kg IV/IO over 20 minutes

or, if already on Phenytoin, give

Phenobarbitone 15-20 mg/kg IV/IO over 10 minutes

CALL ANAESTHETIST

RSI with THIOPENTONE

4 mg/kg IV/IO

Extracted from BCH A&E protocols (Dr S Verma)

**APPENDIX B**

**MOLECULAR ABSORBANT RECIRCULATING SYSTEM (MARS)**

- MARS should be considered in ventilated patients at the point at which renal support would normally be included especially before there is severe encephalopathy or high inotropic requirement.
- Treatment will be given for 8 hours on a daily basis until either:
  1. Liver transplantation
  2. Patient is sufficiently recovered and extubation is considered.

Before initiating MARS:

Contact MARS Technician – See MARS Protocol

1. Liaise with ITU regarding transfer/admission
2. Liaise with Anaesthetists re central catheter insertion
3. If planned admission ensure patient is Nil By Mouth for at least 6 hours and place a nasogastric tube
4. Discuss correction of coagulopathy with ITU and correct thrombocytopenia if platelets <100
5. Ensure availability of large double lumen Vascath, 6.5 for an infant and 11 gauge for a teenager
6. Obtain consent from the family for vascular access for MARS
7. Copies of the ‘Protocol for use of Molecular Absorbant Recirculating System (MARS)’ can be found in the Liver Unit Protocol & Guidelines File on the M-drive.

APPENDIX C

NEONATAL HAEMOCHROMATOSIS

The “Antioxidant cocktail” for rescue treatment of Neonatal Haemochromatosis is as follows:

1. N – acetylcysteine (Acetycysteine) orally:

Dose: 140 mg/kg/day loading dose then 70 mg/kg/day subsequent doses for 17-21 doses.

The total daily dose should be given in three divided doses.

Side effects include: tachycardia, hypotension, rash, mild rise in LFTs.

Oral preparations available are unlicensed and available from Idis World Medicines as:

1. 200mg sachets. Pack size = 20. Brand name = Fluimucil N.
2. 100mg in 5ml syrup. Pack size = 75ml. Brand name is ACC Saft.

The injection solution (200mg / ml) may be used orally at a dilution of 1 in 4.

2. Alpha-tocopheryl acetate (Vitamin E) orally:

Dose: 25 mg/kg/day in two divided doses for 6 weeks.

Side effects include: diarrhoea and abdominal pain.

The suspension is manufactured by Cambridge Laboratory and is available from the wholesalers as

500mg in 5ml. Pack size = 100ml.

1. Desferrioxamine mesilate (Desferal) I.V. or SC. :

Dose: 30 mg/kg/day over 8 hours. Continue until the serum ferritin <500 microgramg/L.

Reconstitute 500 mg vial with 5 ml Water For Injection to give 100mg/ml.

Draw out the total dose for the day and further dilute with a compatible diluent as follows: -

0.3 ml/kg diluted to 10 ml with diluent. Administer this over 8 hours.

(Compatible diluents: glucose 5%, Sodium chloride 0.9%, and glucose- saline combinations).

Note: The iron – Desferrioxamine complex is excreted in the urine, making it orange-red in colour. Side effects include: frequent - local reactions, pain, swelling, headache

Rare - GI disturbance, arrhythmia, hepatic and renal impairment, rashes.

Available as 500mg vials from the wholesalers.

1. Selenium I.V. :

3 microgram/kg/day elemental selenium by continuous intravenous infusion over 24 hours, unless on TPN.

Continue for the length of hospitalisation.

Do not administer undiluted. Further diluted in sodium chloride 0.9% , glucose 5% or glucose 10%

Side effects include: dermatitis; peripheral neuropathy (plasma levels >12.7mcmols/l)

The IV solution has a UK licence with Oxford Nutrition Limited as Sodium Selenite 21.9microcgram/ml. This contains elemental selenium 50microgram/ml. Trade name is Selenase.

1. Alprostadil (Prostaglandin E1, Prostin VR) I.V. :

0.4 microgram/kg/hr increasing to 0.6 microgram/kg/hr over 3-4 hours

Dilute 0.5 ml (250mmicrogram) to 50 ml with sodium chloride 0.9% or glucose 5% to give a 5 microgram/ml solution.

Administer at a rate of 0.08 ml/kg/hr, increasing to 0.12 ml/kg/hr)

Administer into a large vein or through an umbilical artery catheter.

Maximum solution concentration = 20microgram/ml

Side effects include: fever, seizures, flushing, bradycardia, hypotension, apnoea, and reduced platelet

aggregation.

Available as 500microgram/ml solution from the wholesalers, stored in refrigerator.

N.B.: Please note that none of the I.V. drugs are compatible and they need to be

administered through 3 different lines/ lumens

References:

Medicines For Childrens 2003

Guys Paediatric Formulary, 6^th^ ed.

The Harriet Lane Handbook, 16^th^ ed.

BNF 48^th^ ed.

Lexicomp’s Pediatric Dosage Handbook 10^th^ ed.

Sinclair SB, Greig PD, Blendis LM, et al. Biochemical and Clinical response of fulminant viral hepatitis to administration of Prostaglandin E. J Clin Invest 1989;84:1063-9.

Guidelines apply to : All Liver Unit Patients

Originated by : Liver Unit MDTeam

Signed by :

Date of Policy : October 2006

Last update of draft : June 2010

Review Date : November 2010

© Copyright Birmingham Children’s Hospital December 2006

Minor update with Neonatal Haemochromatosis antioxidant cocktail April 2007/gg

Minor update on NAC study details September 2009/

Minor change to Hep E and PCR October 2009

Minor changes to neonatal antibiotic recommended by pharmact Jan2010

Main antibiotic changed to Tazocin. Infusin and neonatal guidelins embedded – feb 2010

Collection of DNA from infants June 2010

ICE requesting replaces blood amounts Sept 2010
