# Supplementary material for: Incidence of Primary Mitochondrial Disease in Children Younger Than 2 Years Presenting With Acute Liver Failure
Source: J Pediatr Gastroenterol Nutr. 2016 Nov 23;63(6):592–7. doi: 10.1097/MPG.0000000000001345 (PMC5113754; doi:10.1097/MPG.0000000000001345)
Supplement: Supplemental Digital Content [file jpga-63-0592-s002.docx]

**Subjects and methods**

All children aged less than 2 years presenting with ALF to a single centre between 2009 and 2011 were included in order to allow at least 5 year follow up. Children were investigated and managed according to an established in house clinical protocol. ALF was defined as the acute onset of liver disease without evidence of chronic liver disease where INR ≥ 2.0, despite parenteral vitamin K and/or where there was hepatic encephalopathy with INR ≥ 1.5. Specific investigations such as cranial MRI and tissue biopsies were at the discretion of the attending clinician and decisions about LT were made by an established multidisciplinary team.

All children had blood taken for sequencing of the *POLG*, *MPV17*, *DGUOK, PEO1, RRM2B* and *TRMU* genes at admission. Where liver or muscle biopsy were undertaken, in addition to conventional histology, samples were sent for mitochondrial studies to the UK Highly Specialised Rare Mitochondrial Disease Service laboratories at Oxford, London or Newcastle upon Tyne. Total mtDNA copy number, relative to nuclear DNA levels, was estimated in muscle and/or liver and compared with age-matched normal controls by validated quantitative real-time PCR assays([1](#_ENREF_1)). MtDNA copy number <30% compared with age-matched controls was classed as mtDNA depletion, 30%-50% as borderline, and >50% as normal. If sufficient tissue was available respiratory chain enzymes were measured by spectrophotometric assays as previously described([2](#_ENREF_2)).

Results are expressed as median and ranges. Groups were compared using the Mann-Whitney U test.

1. Ashley N, O'Rourke A, Smith C, Adams S, Gowda V, Zeviani M, et al. Depletion of mitochondrial DNA in fibroblast cultures from patients with POLG1 mutations is a consequence of catalytic mutations. HumMolGenet. 2008;17(16):2496-506.

2. Hargreaves P, Rahman S, Guthrie P, Taanman JW, Leonard JV, Land JM, et al. Diagnostic value of succinate ubiquinone reductase activity in the identification of patients with mitochondrial DNA depletion. JInheritMetab Dis. 2002;25(1):7-16.
